# Coronary Angiographic Profile and Incidence of Coronary Artery Disease in Patients Undergoing Permanent Pacemaker Implantation for Conduction Abnormalities

**DOI:** 10.7759/cureus.94518

**Published:** 2025-10-13

**Authors:** Suraj Khanal, Mahesh Kumar KS, Basant Kumar, Ravinish Kumar, Mayank Saini

**Affiliations:** 1 Cardiology, Postgraduate Institute of Medical Education & Research, Chandigarh, Chandigarh, IND; 2 Medicine, Postgraduate Institute of Medical Education & Research, Chandigarh, Chandigarh, IND

**Keywords:** conduction abnormality, coronary angiography (cag), coronary artery disease, incidence, permanent pacemaker implantation, permanent pacemaker implantation (ppi)

## Abstract

Background: Symptomatic bradyarrhythmias necessitating permanent pacemaker implantation (PPI) predominantly affect elderly individuals with multiple cardiovascular risk factors. Coronary artery disease (CAD) is a known cause of conduction disturbances, and ischemia affecting the atrioventricular (AV) node or His bundle may present as bradyarrhythmias. However, CAD can be clinically silent, and traditional risk factors often correlate with the presence of coexistent CAD. The prevalence of CAD among pacemaker recipients has been shown to vary widely. Our study aims to assess the incidence of CAD in patients undergoing PPI for conduction abnormalities and identify clinical predictors of CAD in this population.

Aim: This study aims to determine the coronary angiographic profile of patients undergoing permanent pacemaker insertion for conduction abnormalities to assess the incidence and characterization of CAD.

Methods: We conducted a prospective, observational, single-center study at a tertiary care institute, enrolling adult patients with symptomatic bradyarrhythmias (high-grade AV block or sinus node dysfunction) undergoing PPI. We excluded patients with acute coronary syndrome and those with conditions that would preclude invasive procedures. Clinical and demographic data were collected, including a detailed history of coronary risk factors. All patients underwent coronary angiography prior to or during PPI to assess the presence and severity of CAD. CAD was classified as non-obstructive (plaque <50% stenosis) or obstructive (≥50% stenosis). Statistical analysis was performed using chi-square and Student’s t-test, with p<0.05 considered significant.

Results: Among 50 patients (mean age 67 ± 12.5 years), 25 (50%) had normal coronary findings, while 25 (50%) had CAD. Of those with CAD, 11 (22%) had non-obstructive plaques, and 14 (28%) had obstructive CAD. The most common coronary artery involved was the left anterior descending (LAD) artery. Seven patients with obstructive CAD underwent percutaneous coronary intervention (PCI), and seven received optimal medical therapy. Risk factors such as diabetes mellitus (p=0.015) and hypertension (p=0.005) were significantly associated with the presence of obstructive CAD. Echocardiographic findings such as left ventricular ejection fraction (LVEF) and regional wall motion abnormalities (RWMA) were not reliable predictors of CAD presence.

Conclusions: Our study demonstrates that CAD is common among patients undergoing PPI, with a significant proportion having obstructive CAD despite being asymptomatic. Diabetes and hypertension were identified as key risk factors for CAD in this population. The findings suggest that coronary angiography should be considered routinely in high-risk patients undergoing PPI, as it can identify silent CAD and guide therapeutic decisions, ultimately improving prognosis.

## Introduction

Cardiac conduction disturbances may affect the sinoatrial node, atrioventricular (AV) node, or the intraventricular conduction system. Clinical manifestations range from mild weakness to syncope and, in severe cases, sudden death. Persistent or recurrent symptomatic conduction abnormalities usually necessitate permanent pacemaker implantation (PPI). Acute conduction disturbances can arise from myocardial infarction, electrolyte imbalances, drug toxicity (e.g., digitalis), myocarditis (rheumatic or viral), cardiac trauma, or cardiac surgery. Chronic disturbances are often due to degenerative changes, sometimes occurring without other evident myocardial disease, and may also be associated with cardiomyopathy, hypertension, or congenital heart disease.

Furthermore, in some pacemaker recipients, underlying or latent atherosclerotic heart disease may contribute to conduction abnormalities through ischemia of the conduction system [1]. In ischemic cardiomyopathy, sympathetic innervation undergoes remodeling characterized by partial and localized denervation, leading to heterogeneous action potential and conduction responses during sympathetic activation, thereby increasing susceptibility to sudden cardiac death [2].

Symptomatic bradyarrhythmias requiring permanent pacemakers predominantly affect older patients, many of whom have traditional cardiovascular risk factors. While idiopathic degenerative disease of the conduction system (Lenègre’s or Lev’s disease) is a common cause of heart block, ischemia from coronary artery disease (CAD) affecting the AV node or His bundle can also produce conduction disturbances. Moreover, severe bradycardia may mask typical angina, so significant CAD could remain clinically silent. Prior studies have reported a wide range in the prevalence of coexistent CAD among pacemaker recipients (approximately 20%-71%), largely depending on patient risk profile [3-9]. For example, an early series found angiographic CAD in only ~20% of pacemaker patients [4], whereas more recent cohorts enriched with atherosclerotic risk factors demonstrated CAD in about 45%-71% of cases [4-8]. Whether routine coronary evaluation should be pursued in pacemaker candidates without overt ischemic symptoms remains debated. In this context, we undertook a study to determine the incidence and angiographic profile of CAD in patients undergoing PPI for conduction abnormalities and to identify clinical predictors of CAD in this setting.

## Materials and methods

Study design and population

We conducted a single-center prospective observational study at the Postgraduate Institute of Medical Education & Research, a tertiary care institute in Chandigarh, India, from January 2023 to June 2024. Adults (≥18 years) presenting with symptomatic bradyarrhythmias, either high-grade AV block (including complete heart block and Mobitz type II second-degree block) or sinus node dysfunction, who met standard indications for PPI were prospectively enrolled. Patients with acute coronary syndrome at presentation were excluded. Key exclusion criteria were prior pacemaker implantation, acute myocardial infarction requiring emergent pacing support, reversible causes of AV block (e.g., drug-induced or metabolic bradycardia), severe comorbid conditions precluding an invasive procedure, or patient refusal. The study was approved by the institutional ethics committee, and all participants provided informed consent (approval number: IEC-INT/2023/MD-1037).

Clinical assessment

Baseline data collection included detailed history and physical examination with documentation of demographics and coronary risk factors (hypertension, diabetes mellitus, smoking status, dyslipidemia, and family history of CAD). A 12-lead electrocardiogram (ECG) was obtained in all cases to confirm the conduction abnormality, and transthoracic echocardiography was performed to assess left ventricular (LV) function and to look for regional wall motion abnormalities (RWMA) suggestive of prior myocardial infarction.

Coronary angiography

All patients underwent invasive coronary angiography during the index hospitalization, prior to or at the time of pacemaker insertion. Coronary angiograms were performed via a transradial or transfemoral approach and interpreted by two experienced cardiologists blinded to the patients' clinical details. CAD was defined as the presence of any atherosclerotic narrowing in at least one major epicardial coronary artery or its first-order branches. Obstructive CAD was defined as ≥50% diameter stenosis in any vessel, while non-obstructive CAD comprised plaque causing <50% stenosis. Based on the angiographic findings, patients were categorized into three groups: (1) Obstructive CAD: significant stenosis ≥50% in at least one vessel; (2) Non-obstructive CAD: presence of plaque <50% without significant stenosis; (3) Normal coronaries: no angiographic evidence of CAD. For patients with obstructive CAD, the number of diseased vessels was recorded (single-vessel, double-vessel, or triple-vessel disease).

Statistical analysis

Continuous variables are presented as mean ± standard deviation. Categorical variables are summarized as counts and percentages. Groups were compared using the chi-square test (or Fisher’s exact test when expected cell counts were small) for categorical variables and Student’s t-test for continuous variables. In particular, patients with CAD (and specifically those with obstructive CAD) were compared to those without CAD in terms of baseline risk factors (diabetes, hypertension, etc.), clinical presentation (presence of anginal symptoms), and echocardiographic findings (presence of RWMA). Variables with a p-value < 0.05 in univariate comparisons were considered statistically significant. All analyses were performed using IBM SPSS software, version 22.0 (IBM Corp., Armonk, NY), with a two-tailed p < 0.05 considered significant.

## Results

Patient characteristics

A total of 50 patients (mean age 67 ± 12.5 years) underwent PPI for AV or sinoatrial conduction abnormalities. There was a male predominance with 29 (58%) male patients, and the cohort had a high prevalence of coronary risk factors: 27 (54%) had hypertension, 22 (44%) had type 2 diabetes, 12 (30%) had dyslipidemia, and seven (14%) were current or former smokers. Notably, only seven (14%) of the cohort reported any history of chest pain suggestive of angina, and none had experienced a recent acute coronary syndrome (Table 1).

**Table 1:** Patient baseline demographics Data are presented as n (%) and mean ± SD CAD: coronary artery disease; INR: international normalized ratio; TLC: total leukocyte count

Variables	N = 50
Male	29 (58.0)
Age, in years	67± 12.5
Risk factors	
Cardiovascular risk factors	
Diabetes	22 (44)
Hypertension	27 (54)
Dyslipidemia	12 (30.0)
Smoker	7 (14)
Past history of CAD	3 (6)
Family history of conduction abnormalities	1 (2)
Non-cardiovascular risk factors	
Hypothyroidism	5 (12.5)
Symptoms	
Presyncope	34 (68)
Syncope	15 (30)
Dyspnea	19 (38)
Chest pain	7 (14)
Palpitations	3 (6)
Laboratory parameters	
Hemoglobin (g/dl)	12.33 ± 1.65
TLC (K)	8.53 ± 2.98
Platelets(k)	177.50 ± 82.31
Sodium	132.90 ± 13.5
Potassium	4.40 ± 0.61
Urea	43.98 ± 21.53
Creatinine	1.24 ± 1.19
INR	1.01 ± 0.13

The majority of patients (28, 56%) presented with complete heart block, 10 (20%) had symptomatic sick sinus syndrome, about three (6%) had advanced second-degree AV block (Mobitz II), and three (6%) had trifascicular block. The majority of patients had symptoms attributable to bradycardia, such as presyncope in 34 (68%) patients or syncope in 15 (30%) patients (Table 2).

**Table 2:** Electrocardiographic (ECG) findings and conduction abnormalities in study population Data are presented as n (%) AV: atrioventricular; CHB: complete heart block

ECG findings	Frequency (n)	Percentage (%)
First-degree AV block	2	4.0
2:1 AV block with intermittent CHB	3	6.0
3:1 AV block	2	4.0
CHB	28	56.0
Junctional bradycardia	2	4.0
Sick sinus syndrome	10	20.0
Trifascicular block	3	6.0
Total (n)	50	100.0

Baseline echocardiography showed a preserved LV ejection fraction (LVEF>50%) and no RWMA in 40 (80%) patients. RWMA was demonstrated in 10 (20%) patients. Regarding echocardiographic findings, patients were categorized into two groups based on LVEF and the presence of RWMA. Of the 40 (80%) patients with normal LVEF (50-60%) and no RWMA, 20 (40%) had no CAD, 11 (22%) had non-obstructive CAD, and 9 (18%) had significant obstructive CAD. Of the 10 (20%) patients with reduced LVEF (≤50%) and RWMA, five (8%) had obstructive CAD. These findings suggest that echocardiographic findings alone, such as LVEF and RWMA, were not reliable predictors of CAD presence, highlighting the need for more comprehensive diagnostic methods, like coronary angiography, to assess CAD in this population (Table 3).

**Table 3:** Left ventricular function assessment stratified by coronary angiographic findings Data are presented as n (%) CAD: coronary artery disease; EF: ejection fraction; RWMA: regional wall motion abnormality

Variables	Angiographic CAD			Total N (%)
	No CAD	Obstructive CAD	Non-obstructive CAD	
Normal EF, No RWMA				
50%-60% EF	20 (40)	9 (18)	11 (22)	40 (80)
EF ≤50%, with RWMA				
≤50%	5 (10)	5 (8)	0 (0)	10 (20)
Total	25 (50)	14 (28)	11 (22)	50 (100)

Incidence and extent of CAD

**Figure 1:**
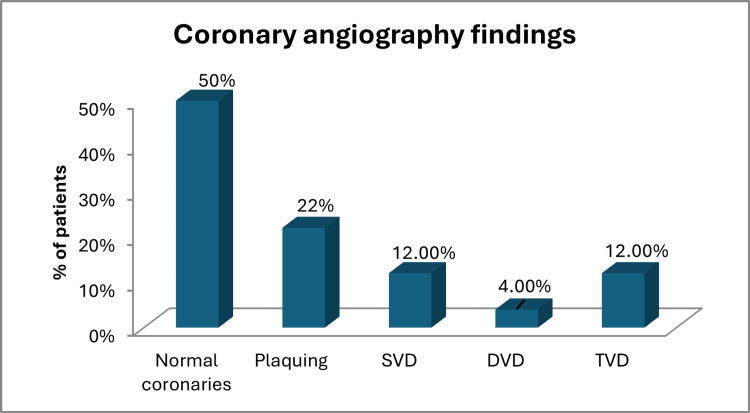
Distribution of coronary angiography findings in patient population DVD: double vessel disease; SVD: single vessel disease; TVD: triple vessel disease

Among the 50 patients undergoing PPI, coronary angiography was performed to assess the presence of CAD. Of the 50 patients, 25 (50%) had normal coronary findings, while 25 (50%) had CAD. Among those with CAD, 11 (22%) had non-obstructive plaques and 14 (28%) had obstructive CAD. Of the 14 (28%) patients with obstructive CAD, six (12%) had single-vessel disease, two (4%) had double-vessel disease, and six (12%) had triple-vessel disease (Table 4, Figure 1).

**Table 4:** Distribution of coronary angiographic findings Data are presented as n (%) DVD: double vessel disease; SVD: single vessel disease; TVD: triple vessel disease

Coronary angiography findings	Frequency (n)	Percentage (%)
Normal coronaries	25	50
Plaquing	11	22
SVD	6	12.00
DVD	2	4.00
TVD	6	12.00
Total	50	100.00

The left anterior descending (LAD) artery was most commonly involved, while coronary dominance was predominantly right-sided (38, or 76%), with five (10%) left-dominant and seven (14%) co-dominant circulations. Regarding treatment, seven (14%) patients with significant CAD underwent PCI, while seven (14%) others were managed with optimal medical therapy.

Risk factor correlations

In our study, we explored the relationship between various risk factors and the presence of obstructive CAD. Among the 50 patients, 40 (80%) had one or more comorbidities, with 30 (60%) having multiple comorbidities. We found that the presence of significant CAD was significantly correlated with diabetes mellitus (p=0.015) and hypertension (p=0.005). However, no significant correlation was found between CAD and other factors such as smoking, alcohol use, hypothyroidism, dyslipidemia, past history of acute coronary syndrome, family history of CAD, or chronic kidney disease (CKD). The findings suggest that diabetes and hypertension are strong predictors of CAD in this population, whereas other risk factors did not show significant associations (Table 5).

**Table 5:** Association between cardiovascular risk factors and obstructive CAD Data are presented as n (%). Chi-square analysis was performed for categorical variables with significance levels indicated by *p < 0.05 and **p < 0.01. ACS: acute coronary syndrome; ASCVD: atherosclerotic cardiovascular disease; CAD: coronary artery disease; CKD: chronic kidney disease; CSA: chronic stable angina; ICMP: ischemic cardiomyopathy; PCI: percutaneous coronary intervention

Risk factors	Obstructive CAD			Chi-Square	p-value
	Yes	No	Total		
Cardiovascular risk-factors					
Diabetes mellitus	10 (71.4)	12 (33.3)	22 (44.0)	5.937	0.015*
Hypertension	12 (85.7)	15 (41.7)	27 (54.0)	7.873	0.005**
Smoking	3 (21.4)	4 (11.1)	7 (14.0)	0.891	0.345
Dyslipidemia	1 (7.1)	1 (2.8)	2 (4.0)	0.500	0.479
Past history of ACS	2 (14.3)	1 (2.8)	3 (6.0)	2.367	0.124
Past history of ASCVD (CAD (CSA, USA, ICMP)	2 (14.3)	3 (8.3)	5 (10.0)	0.397	0.529
Past history of PCI	1 (7.1)	1 (2.8)	2 (4.0)	0.500	0.479
Family history of CAD	0 (0.0)	2 (5.6)	2 (4.0)	0.810	0.368
Non-cardiovascular risk-factors					
Alcohol usage	3 (21.4)	7 (19.4)	10 (20.0)	0.025	0.875
Hypothyroidism	1 (7.1)	3 (8.3)	4 (8.0)	0.019	0.889
Family history of conduction abnormalities	0 (0.0)	1 (2.8)	1 (2.0)	0.397	0.529
CKD	1 (7.1)	0 (0.0)	1 (2.0)	2.624	0.105
Total	14 (100)	36 (100)	50 (100)	-	-

Symptoms and echocardiographic findings 

In our study, we assessed the correlation between presenting symptoms and the presence of obstructive CAD. Symptoms such as syncope, presyncope, shortness of breath, chest pain, dizziness, and palpitations were reported, but no significant correlation was found between these symptoms and the presence of obstructive CAD (Table 6).

**Table 6:** Clinical symptom presentation in patients with and without obstructive CAD CAD: coronary artery disease

Symptoms	Obstructive CAD			Chi-square	p-value
	Yes	No	Total		
Syncope	3 (21.4)	12 (33.3)	15 (30.0)	0.680	0.409
Presyncope	1 (7.1)	5 (13.9)	6 (12.0)	0.434	0.51
Shortness of breath	7 (50)	12 (33.3)	19 (38.0)	1.188	0.276
Chest pain	2 (14.3)	5 (13.9)	7 (14.0)	0.001	0.971
Dizziness	9 (64.3)	19 (52.8)	28 (56.0)	0.542	0.462
Palpitations	1 (7.1)	2 (5.6)	3 (6.0)	0.045	0.832
Total	14 (100)	36 (100)	50 (100.0)	-	-

## Discussion

In our cohort of patients undergoing PPI for conduction disturbances, the incidence of coexisting CAD was approximately 50% on coronary angiography. About half of these CAD-positive patients had obstructive CAD (angiographic stenoses ≥50%), while the rest had non-obstructive coronary plaques. Patients with CAD tended to be older (most were in the seventh decade of life), and traditional cardiovascular risk factors were prevalent: notably, diabetes mellitus was significantly associated with the presence of CAD in our study population (present in ~60% of CAD patients vs. ~24% without CAD), whereas hypertension, although common overall, did not show a statistically significant difference between those with and without CAD. There was a male predominance in the CAD group (65% of CAD patients were male), although this did not reach significance (p≈0.06).

Symptomatology and echocardiographic findings were poor discriminators of CAD status: most patients, with or without CAD, presented with syncope or dyspnea rather than angina, and the baseline characteristics and presenting symptoms did not differ significantly between patients with and without angiographic CAD. Importantly, a substantial subset of individuals with normal LVEF and no RWMA on echocardiogram were found to have occult CAD on angiography, underscoring that the absence of ischemic signs on non-invasive evaluation did not exclude significant coronary disease. Our findings align with prior studies reporting a high prevalence of silent CAD in patients requiring PPI, especially among those with risk factors: published series have documented coexistent CAD in roughly 20%-50% of unselected pacemaker patients and up to 70% or more in high-risk subgroups. [6,10].

The major artery involved in our study was the LAD artery. An Indian study by Mohammad S. Ali et al. showed that the major arteries involved are the right coronary artery (RCA) and LAD, respectively [11]. A similar study by Rajendran V et al. revealed that 34.4% of their study population had coexisting significant CAD. The major artery involved was the RCA [12]. Similarly, other investigators have identified advanced age, male sex, hypertension, and diabetes as significant predictors of CAD in this context [1,6,11]. In our study, all patients proceeded to PPI regardless of angiographic results, but the identification of CAD had important management implications. Those with non-obstructive or moderate lesions received optimized medical therapy for CAD, whereas a minority (≈7% of the total cohort) had critical stenoses that prompted revascularization, most commonly PCI, with one case undergoing coronary bypass surgery. The clinical relevance of detecting CAD in patients undergoing PPI is highlighted by the potential to improve outcomes through appropriate therapy: coexistent CAD can adversely affect long-term prognosis and may be aggravated by pacing-related factors (loss of atrioventricular synchrony and altered ventricular activation) if left unrecognized [13,14]. Moreover, knowledge of significant CAD (especially if associated with even mildly reduced ventricular function) can influence device selection. For instance, clinicians might consider upfront implantation of a cardiac resynchronization, capable pacemaker, or defibrillator in a patient with bradyarrhythmia who has concomitant ischemic heart disease to mitigate pacing-induced heart failure and arrhythmic risk. Therefore, our study underscores the importance of considering routine coronary angiographic evaluation in high-risk candidates for PPI, as diagnosing and managing concomitant CAD (through revascularization or intensive medical therapy) is likely to confer additional prognostic benefit beyond that provided by the pacemaker itself.

Limitations of the study

This single-center study with a small sample size is prone to Berksonian bias and lacks a control group, limiting generalizability. It included only patients requiring PPI, excluding asymptomatic or less severe conduction abnormalities. The impact of revascularization on outcomes was not assessed. Larger, multicentric prospective studies with long-term follow-up are needed to validate these findings and clarify the relationship between conduction abnormalities, CAD, and treatment outcomes.

## Conclusions

The identification of significant CAD in patients with normal echocardiographic findings underscores the limitations of non-invasive assessment and highlights the insidious nature of silent ischemia, where traditional symptom-based screening approaches fail to detect clinically relevant coronary disease. Diabetes mellitus and hypertension emerged as the most significant predictors of obstructive CAD, validating their utility in risk stratification for this patient cohort, while echocardiographic parameters proved unreliable in predicting coronary disease presence. The therapeutic implications are substantial, as routine coronary angiography enabled appropriate management strategies with equal proportions receiving percutaneous intervention and optimal medical therapy, while the detection of coexistent CAD has important prognostic implications that may influence device selection strategies and long-term outcomes. Therefore, our findings support implementing routine coronary angiographic evaluation in high-risk pacemaker candidates, particularly those with diabetes and hypertension, as this proactive approach can identify clinically silent but significant CAD, guide evidence-based therapeutic decisions, and ultimately improve overall cardiovascular prognosis through integrated coronary disease management with device therapy in this vulnerable population.
